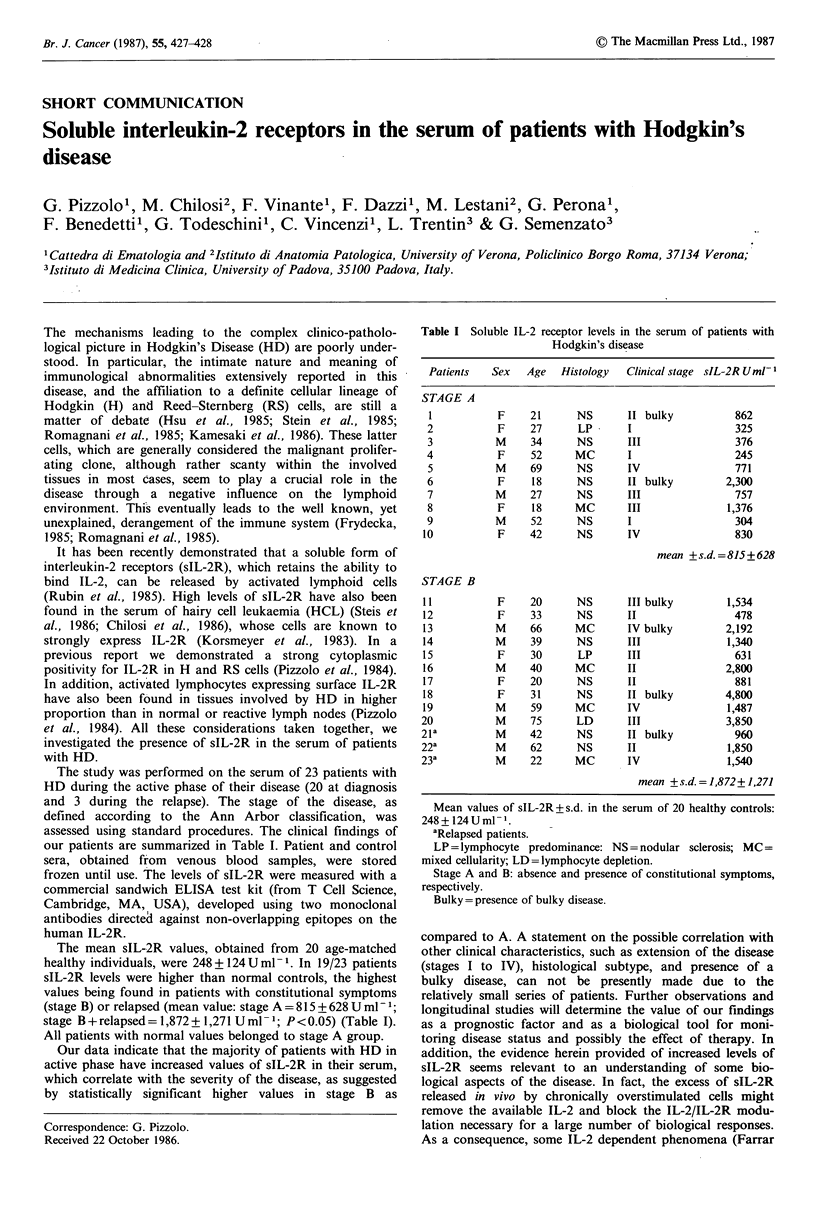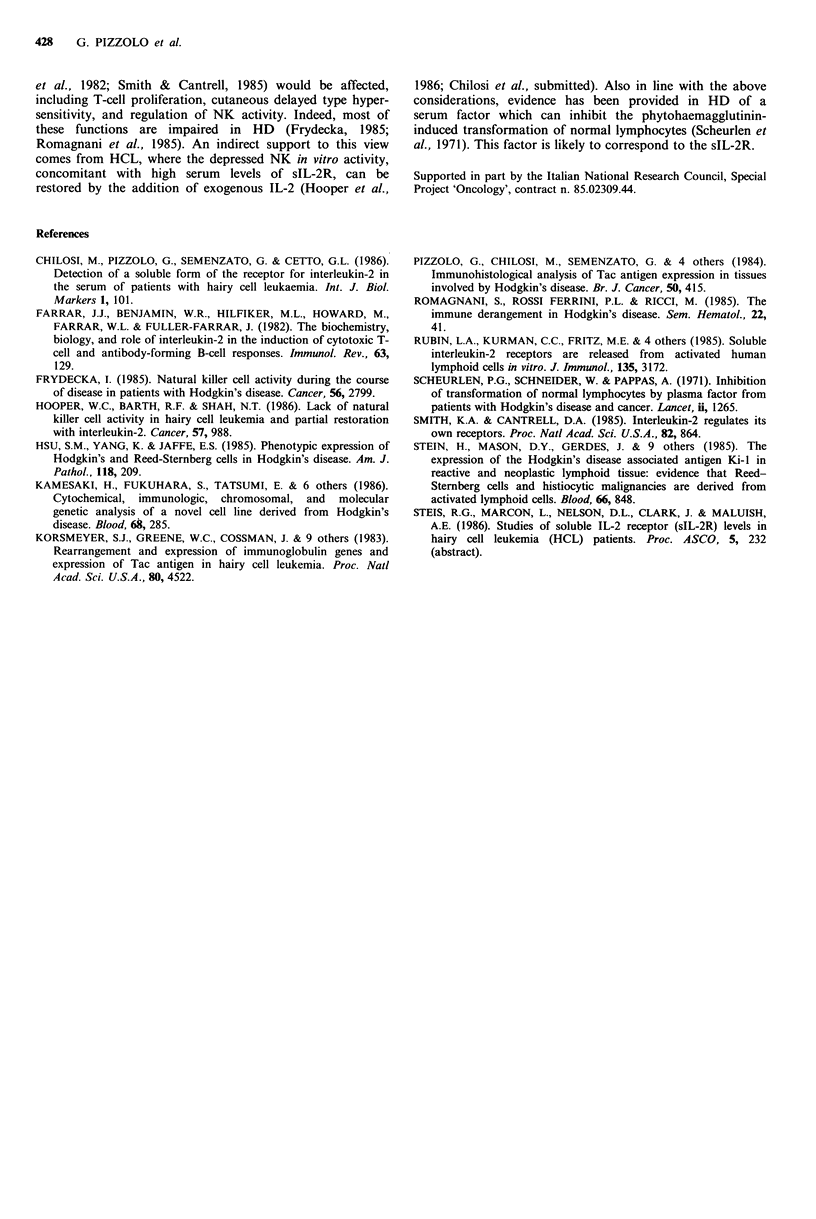# Soluble interleukin-2 receptors in the serum of patients with Hodgkin's disease.

**DOI:** 10.1038/bjc.1987.83

**Published:** 1987-04

**Authors:** G. Pizzolo, M. Chilosi, F. Vinante, F. Dazzi, M. Lestani, G. Perona, F. Benedetti, G. Todeschini, C. Vincenzi, L. Trentin


Br. J. Cancer (1987), 55, 427–428 © The Macmillan Press Ltd., 1987

SHORT COMMUNICATION

### Soluble interleukin-2 receptors in the serum of patients with Hodgkin's disease

G. Pizzolo[1], M. Chilosi[2], F. Vinante[1], F. Dazzi[1], M. Lestani[2], G. Perona[1], F. Benedetti[1], G. Todeschini[1], C. Vincenzi[1], L. Trentin[3] & G. Semenzato[3]

[1]*Cattedra di Ematologia and* [2]*Istituto di Anatomia Patologica, University of Verona, Policlinico Borgo Roma, 37134 Verona;* [3]*Istituto di Medicina Clinica, University of Padova, 35100 Padova, Italy.*

The mechanisms leading to the complex clinico-pathological picture in Hodgkin's Disease (HD) are poorly understood. In particular, the intimate nature and meaning of immunological abnormalities extensively reported in this disease, and the affiliation to a definite cellular lineage of Hodgkin (H) and Reed–Sternberg (RS) cells, are still a matter of debate (Hsu *et al.*, 1985; Stein *et al.*, 1985; Romagnani *et al.*, 1985; Kamesaki *et al.*, 1986). These latter cells, which are generally considered the malignant proliferating clone, although rather scanty within the involved tissues in most cases, seem to play a crucial role in the disease through a negative influence on the lymphoid environment. This eventually leads to the well known, yet unexplained, derangement of the immune system (Frydecka, 1985; Romagnani *et al.*, 1985).

It has been recently demonstrated that a soluble form of interleukin-2 receptors (sIL-2R), which retains the ability to bind IL-2, can be released by activated lymphoid cells (Rubin *et al.*, 1985). High levels of sIL-2R have also been found in the serum of hairy cell leukaemia (HCL) (Steis *et al.*, 1986; Chilosi *et al.*, 1986), whose cells are known to strongly express IL-2R (Korsmeyer *et al.*, 1983). In a previous report we demonstrated a strong cytoplasmic positivity for IL-2R in H and RS cells (Pizzolo *et al.*, 1984). In addition, activated lymphocytes expressing surface IL-2R have also been found in tissues involved by HD in higher proportion than in normal or reactive lymph nodes (Pizzolo *et al.*, 1984). All these considerations taken together, we investigated the presence of sIL-2R in the serum of patients with HD.

The study was performed on the serum of 23 patients with HD during the active phase of their disease (20 at diagnosis and 3 during the relapse). The stage of the disease, as defined according to the Ann Arbor classification, was assessed using standard procedures. The clinical findings of our patients are summarized in Table I. Patient and control sera, obtained from venous blood samples, were stored frozen until use. The levels of sIL-2R were measured with a commercial sandwich ELISA test kit (from T Cell Science, Cambridge, MA, USA), developed using two monoclonal antibodies directed against non-overlapping epitopes on the human IL-2R.

The mean sIL-2R values, obtained from 20 age-matched healthy individuals, were $248 \pm 124\,U\,ml^{-1}$. In 19/23 patients sIL-2R levels were higher than normal controls, the highest values being found in patients with constitutional symptoms (stage B) or relapsed (mean value: stage $A = 815 \pm 628\,U\,ml^{-1}$; stage B + relapsed $= 1{,}872 \pm 1{,}271\,U\,ml^{-1}$; $P < 0.05$) (Table I). All patients with normal values belonged to stage A group.

Our data indicate that the majority of patients with HD in active phase have increased values of sIL-2R in their serum, which correlate with the severity of the disease, as suggested by statistically significant higher values in stage B as compared to A. A statement on the possible correlation with other clinical characteristics, such as extension of the disease (stages I to IV), histological subtype, and presence of a bulky disease, can not be presently made due to the relatively small series of patients. Further observations and longitudinal studies will determine the value of our findings as a prognostic factor and as a biological tool for monitoring disease status and possibly the effect of therapy. In addition, the evidence herein provided of increased levels of sIL-2R seems relevant to an understanding of some biological aspects of the disease. In fact, the excess of sIL-2R released *in vivo* by chronically overstimulated cells might remove the available IL-2 and block the IL-2/IL-2R modulation necessary for a large number of biological responses. As a consequence, some IL-2 dependent phenomena (Farrar

**Table I** Soluble IL-2 receptor levels in the serum of patients with Hodgkin's disease

| *Patients* | *Sex* | *Age* | *Histology* | *Clinical stage* | *sIL-2R U ml$^{-1}$* |
|---|---|---|---|---|---|
| *STAGE A* | | | | | |
| 1 | F | 21 | NS | II bulky | 862 |
| 2 | F | 27 | LP | I | 325 |
| 3 | M | 34 | NS | III | 376 |
| 4 | F | 52 | MC | I | 245 |
| 5 | M | 69 | NS | IV | 771 |
| 6 | F | 18 | NS | II bulky | 2,300 |
| 7 | M | 27 | NS | III | 757 |
| 8 | F | 18 | MC | III | 1,376 |
| 9 | M | 52 | NS | I | 304 |
| 10 | F | 42 | NS | IV | 830 |
| | | | | | *mean ±s.d. = 815±628* |
| *STAGE B* | | | | | |
| 11 | F | 20 | NS | III bulky | 1,534 |
| 12 | F | 33 | NS | II | 478 |
| 13 | M | 66 | MC | IV bulky | 2,192 |
| 14 | M | 39 | NS | III | 1,340 |
| 15 | F | 30 | LP | III | 631 |
| 16 | M | 40 | MC | II | 2,800 |
| 17 | F | 20 | NS | II | 881 |
| 18 | F | 31 | NS | II bulky | 4,800 |
| 19 | M | 59 | MC | IV | 1,487 |
| 20 | M | 75 | LD | III | 3,850 |
| 21[a] | M | 42 | NS | II bulky | 960 |
| 22[a] | M | 62 | NS | II | 1,850 |
| 23[a] | M | 22 | MC | IV | 1,540 |
| | | | | | *mean ±s.d. = 1,872±1,271* |

Mean values of sIL-2R ± s.d. in the serum of 20 healthy controls: $248 \pm 124\,U\,ml^{-1}$.

[a]Relapsed patients.

LP = lymphocyte predominance: NS = nodular sclerosis; MC = mixed cellularity; LD = lymphocyte depletion.

Stage A and B: absence and presence of constitutional symptoms, respectively.

Bulky = presence of bulky disease.

Correspondence: G. Pizzolo.
Received 22 October 1986.

*et al.*, 1982; Smith & Cantrell, 1985) would be affected, including T-cell proliferation, cutaneous delayed type hypersensitivity, and regulation of NK activity. Indeed, most of these functions are impaired in HD (Frydecka, 1985; Romagnani *et al.*, 1985). An indirect support to this view comes from HCL, where the depressed NK *in vitro* activity, concomitant with high serum levels of sIL-2R, can be restored by the addition of exogenous IL-2 (Hooper *et al.*, 1986; Chilosi *et al.*, submitted). Also in line with the above considerations, evidence has been provided in HD of a serum factor which can inhibit the phytohaemagglutinin-induced transformation of normal lymphocytes (Scheurlen *et al.*, 1971). This factor is likely to correspond to the sIL-2R.

Supported in part by the Italian National Research Council, Special Project 'Oncology', contract n. 85.02309.44.

### References

CHILOSI, M., PIZZOLO, G., SEMENZATO, G. & CETTO, G.L. (1986). Detection of a soluble form of the receptor for interleukin-2 in the serum of patients with hairy cell leukaemia. *Int. J. Biol. Markers* **1**, 101.

FARRAR, J.J., BENJAMIN, W.R., HILFIKER, M.L., HOWARD, M., FARRAR, W.L. & FULLER-FARRAR, J. (1982). The biochemistry, biology, and role of interleukin-2 in the induction of cytotoxic T-cell and antibody-forming B-cell responses. *Immunol. Rev.*, **63**, 129.

FRYDECKA, I. (1985). Natural killer cell activity during the course of disease in patients with Hodgkin's disease. *Cancer*, **56**, 2799.

HOOPER, W.C., BARTH, R.F. & SHAH, N.T. (1986). Lack of natural killer cell activity in hairy cell leukemia and partial restoration with interleukin-2. *Cancer*, **57**, 988.

HSU, S.M., YANG, K. & JAFFE, E.S. (1985). Phenotypic expression of Hodgkin's and Reed-Sternberg cells in Hodgkin's disease. *Am. J. Pathol.*, **118**, 209.

KAMESAKI, H., FUKUHARA, S., TATSUMI, E. & 6 others (1986). Cytochemical, immunologic, chromosomal, and molecular genetic analysis of a novel cell line derived from Hodgkin's disease. *Blood*, **68**, 285.

KORSMEYER, S.J., GREENE, W.C., COSSMAN, J. & 9 others (1983). Rearrangement and expression of immunoglobulin genes and expression of Tac antigen in hairy cell leukemia. *Proc. Natl Acad. Sci. U.S.A.*, **80**, 4522.

PIZZOLO, G., CHILOSI, M., SEMENZATO, G. & 4 others (1984). Immunohistological analysis of Tac antigen expression in tissues involved by Hodgkin's disease. *Br. J. Cancer*, **50**, 415.

ROMAGNANI, S., ROSSI FERRINI, P.L. & RICCI, M. (1985). The immune derangement in Hodgkin's disease. *Sem. Hematol.*, **22**, 41.

RUBIN, L.A., KURMAN, C.C., FRITZ, M.E. & 4 others (1985). Soluble interleukin-2 receptors are released from activated human lymphoid cells *in vitro*. *J. Immunol.*, **135**, 3172.

SCHEURLEN, P.G., SCHNEIDER, W. & PAPPAS, A. (1971). Inhibition of transformation of normal lymphocytes by plasma factor from patients with Hodgkin's disease and cancer. *Lancet*, **ii**, 1265.

SMITH, K.A. & CANTRELL, D.A. (1985). Interleukin-2 regulates its own receptors. *Proc. Natl Acad. Sci. U.S.A.*, **82**, 864.

STEIN, H., MASON, D.Y., GERDES, J. & 9 others (1985). The expression of the Hodgkin's disease associated antigen Ki-1 in reactive and neoplastic lymphoid tissue: evidence that Reed–Sternberg cells and histiocytic malignancies are derived from activated lymphoid cells. *Blood*, **66**, 848.

STEIS, R.G., MARCON, L., NELSON, D.L., CLARK, J. & MALUISH, A.E. (1986). Studies of soluble IL-2 receptor (sIL-2R) levels in hairy cell leukemia (HCL) patients. *Proc. ASCO*, **5**, 232 (abstract).